# The role of autoimmune antibodies to predict secondary autoimmunity in patients with relapsing-remitting multiple sclerosis treated with alemtuzumab: A nationwide prospective survey

**DOI:** 10.3389/fneur.2023.1137665

**Published:** 2023-03-16

**Authors:** Sofia Sandgren, Lenka Novakova, Markus Axelsson, Firoozeh Amirbeagi, Ingrid Kockum, Tomas Olsson, Clas Malmestrom, Jan Lycke

**Affiliations:** ^1^Department of Clinical Neuroscience, Institute of Neuroscience and Physiology at Sahlgrenska Academy, University of Gothenburg, Gothenburg, Sweden; ^2^Laboratory for Clinical Immunology, Sahlgrenska Academy, University of Gothenburg, Gothenburg, Sweden; ^3^Department of Clinical Neuroscience, Karolinska Institute, Stockholm, Sweden; ^4^Center for Molecular Medicine, Karolinska University Hospital, Stockholm, Sweden

**Keywords:** Graves' disease, secondary autoimmunity, alemtuzumab (Lemtrada), multiple sclerosis, autoimmune antibodies, autoimmune thyroid disease (AITD)

## Abstract

**Background:**

Alemtuzumab (ALZ) is an immune reconstitution therapy for treating relapsing-remitting multiple sclerosis (RRMS). However, ALZ increases the risk of secondary autoimmune diseases (SADs).

**Objective:**

We explored whether the detection of autoimmune antibodies (auto-Abs) could predict the development of SADs.

**Methods:**

We included all patients with RRMS in Sweden who initiated ALZ treatment (*n* = 124, 74 female subjects) from 2009 to 2019. The presence of auto-Abs was determined in plasma samples obtained at the baseline and at 6, 12, and 24 months of follow-up, as well as in a subgroup of patients (*n* = 51), it was determined in plasma samples obtained at the remaining 3-month intervals up to 24 months. Monthly blood tests, urine tests, and the assessment of clinical symptoms were performed for monitoring safety including that of SADs.

**Results:**

Autoimmune thyroid disease (AITD) developed in 40% of patients, within a median follow-up of 4.5 years. Thyroid auto-Abs were detected in 62% of patients with AITD. The presence of thyrotropin receptor antibodies (TRAbs) at the baseline increased the risk of AITD by 50%. At 24 months, thyroid auto-Abs were detected in 27 patients, and 93% (25/27) developed AITD. Among patients without thyroid auto-Abs, only 30% (15/51) developed AITD (*p* < 0.0001). In the subgroup of patients (*n* = 51) with more frequent sampling for auto-Abs, 27 patients developed ALZ-induced AITD, and 19 of them had detectable thyroid auto-Abs prior to the AITD onset, with a median interval of 216 days. Eight patients (6.5%) developed non-thyroid SAD, and none had detectable non-thyroid auto-Abs.

**Conclusion:**

We conclude that monitoring thyroid auto-Abs, essentially TRAbs, may improve the surveillance of AITD associated with ALZ treatment. The risk for non-thyroid SADs was low, and monitoring non-thyroid auto-Abs did not seem to provide any additional information for predicting non-thyroid SADs.

## Introduction

Alemtuzumab (ALZ) is an immune reconstitution therapy for treating relapsing-remitting multiple sclerosis (RRMS). After two ALZ courses of 5 and 3 days, administered 12 months apart, new ALZ infusions can be administered at signs of new inflammatory disease activity. Each ALZ infusion causes a pan-lymphocyte depletion, where all circulating lymphocytes are reduced within a few days. After this pronounced lymphocyte reduction, T and B lymphocytes repopulate, which can contribute to a sustainable effect (1, 2). In clinical practice, ALZ is mostly used as a second- or third-line treatment for RRMS, and this treatment is less restricted, in terms of patient age and comorbidities, than the ALZ treatments described in the clinical trial program of ALZ (3–5). Furthermore, these studies (3–5) excluded patients with clinically significant autoimmunity or thyrotropin receptor antibodies (TRAbs) at the baseline. Hence, “real-world” patients treated with ALZ differ significantly from the RRMS populations that were included in the ALZ treatment trials.

Alemtuzumab is considered a highly effective disease-modifying therapy for RRMS. However, its utility is limited because it increases the risk of infections, infusion-related side effects, and in particular, secondary autoimmune diseases (SADs). Among patients treated with ALZ, 30–48% develop SADs, and the incidence peaks after 2–3 years (3–8). The most common SADs are autoimmune thyroid disease (AITD), immune thrombocytopenia (ITP), and autoimmune nephropathies. In the pivotal ALZ studies CARE-MS I and II (4, 5) and their extensions (7, 8), the prevalence of AITD was 20 and 40%, ITP was 1 and 3%, and autoimmune nephropathies were 0.3 and 0.3%, respectively. A range of other rare disorders has also been reported in patients treated with ALZ, including neutropenia, hemolytic anemia, and autoimmune hepatitis (4, 5, 7–9). A recent safety analysis of the ALZ clinical trial program found that post-ALZ treatment SADs were similar among patients with preexisting non-multiple sclerosis (MS) autoimmunity (35.4%) and those without it (35.3%), and these SADs were serious in 8.8 and 9.1% of cases, respectively (10). In 2019, ALZ underwent a procedure under Article 20 of Regulation (EC) No 726/2004 resulting from pharmacovigilance data, which led to label change effective from January 2020 (11). However, since the beginning of the last year, the restrictions on the use of ALZ as first-line therapy became less strong. According to the last summary of the product characteristics update from the European Medicines Agency, ALZ can now be used to treat RRMS if the disease is highly active despite treatment with at least one disease-modifying treatment or in patients with rapidly evolving severe RRMS (12). ALZ must also no longer be used in patients with certain heart, circulation, or bleeding disorders or in patients who have autoimmune disorders other than MS.

The pathogenic mechanism for post-ALZ SADs remains unclear, but SADs appear to occur during and after a phase of immune reconstitution, following lymphopenia. Similar inductions of autoimmunity have been reported after antiretroviral therapy in patients with human immunodeficiency virus, and in patients after bone marrow transplantation (13, 14). Although the association between lymphopenia and autoimmunity is well recognized, most patients with lymphopenia do not develop autoimmunity during lymphocyte recovery; hence, additional factors seem to be involved in this process.

It was previously suggested that after ALZ treatment, SADs occurred in patients with increased pre-treatment serum interleukin-21 levels, which gave rise to elevated T-cell apoptosis and cell cycling (15). However, those findings could not be verified in larger prospective cohorts with currently available interleukin-21 immunosorbent assays (16). A small retrospective study on the use of custom-made TRAb assays demonstrated that measuring the levels of TRAb at the baseline and antibodies (Abs) against thyroid peroxidase (TPOAbs) could predict an increased risk of AITD after ALZ treatment (17). Furthermore, a prospective study employed standard tests for TPOAbs and thyroglobulin Abs (TgAbs); they demonstrated that the detection of these autoimmune Abs (auto-Abs) at the baseline indicated an increased risk of developing clinically manifest AITD after ALZ treatment (18). Recently published clinical trial data support a risk of SAD among 51–67% and 26–35% of patients with respective without TPOAbs at the baseline (10). However, lymphocyte repopulation dynamics have not been shown to predict SADs occurrence after ALZ treatment (19). A recent study presented findings supporting that the risk of SADs in predisposed ALZ-treated patients was linked to ALZ-specific immune repertoire changes, including the restriction of T- and B-cell repertoire, reduced thymopoiesis, homeostatic proliferation, and disparate dynamics of clonal T- and B-cell expansion (20). Furthermore, previous treatment and the treatment sequence impact the efficacy and safety profile of ALZ (21).

The present observational, prospective, nationwide study included a “real-world” Swedish cohort of patients with RRMS that were treated with ALZ. We explored whether we, in line with previous studies in the field, could confirm that the detection of auto-Abs at the baseline (i.e., before initiating ALZ) or during ALZ treatment could predict the development of SADs. This prediction could improve monitoring safety and might have implications for therapeutic decisions.

## Materials and methods

### Study population

This prospective observational, study included all patients with RRMS in Sweden that initiated ALZ treatment between February 2009 and January 2019, of which single patients participated in CARE-MS I (4) and its extension study TOPAZ (22). Patients received 60 mg ALZ intravenous (i.v.) over 5 days, and after 1 year, 36 mg ALZ i.v. infused over 3 days. New 3-day courses of 36 mg ALZ were administered when a clinical relapse occurred and/or new or enlarging lesions were detected with magnetic resonance imaging (MRI). Patients that received at least one ALZ infusion were included in the study. We recruited patients from the MS Center of Sahlgrenska University Hospital, Gothenburg, and patients from the “Immunomodulation and Multiple Sclerosis Epidemiology” study, a drug monitoring registry of disease-modifying therapies for MS in Sweden, at the Karolinska Institute, Stockholm, Sweden.

### Sampling for auto-Abs detection

Plasma samples were obtained at the baseline, and at 6, 12, and 24 months for all included patients, and for a subgroup of patients (*n* = 51), plasma samples were obtained at the baseline and then every 3 months up to 24 months. All samples were stored at −80°C until analyzed. Auto-Abs analyses were performed at the Clinical Immunology Laboratory, Sahlgrenska University Hospital, Gothenburg, Sweden.

### Auto-Abs analyses

TgAbs, TPOAbs, and anti-glomerular basement membrane Abs (GBMAbs) were analyzed with an indirect enzyme immunoassay using an Alegria^®^ analyzer (Orgentec Diagnostica, Mainz, Germany). Assays were performed according to the laboratory's accreditation, with an 8% coefficient of variation. TRAbs were analyzed with an enzyme-linked immunosorbent assay (ELISA) kit (ElisaRSR^TM^ TRAb Fast^TM^, RSR Limited, Cardiff, United Kingdom), according to the manufacturer's instructions, with a 7.7% coefficient of variation. Glutamic acid decarboxylase Abs (GADAbs) were analyzed with an anti-GAD ELISA (IgG) assay, specific for the GAD65 isoform (Euroimmun, Lübeck, Germany). Assays were performed, according to clinical standard operating procedures, with a 4.7% coefficient of variation. Antinuclear Abs (ANAs), smooth muscle Abs (SMAs), antimitochondrial Abs (AMAs, M2), and liver kidney microsomal antigens (LKMs) were analyzed with indirect immunofluorescence (Liver mosaic 8, Euroimmun, Lübeck, Germany). Thus, patient plasma samples were diluted in a ratio of 1:100 in phosphate-buffered saline and incubated on mosaic slides in wells that contained Hep2 cells, VSM47 cells transfected with f-actin, or sections of the liver/kidney/stomach (rat) or the liver (monkey). The slides were qualitatively evaluated with an Immunofluorescence-Olympus BX-41 Phase Contrast microscope (Olympus, Shinjuku, Tokyo prefecture, Japan), according to the manufacturer's instructions. All auto-Abs analyses were performed at the Clinical Immunology Laboratory of Sahlgrenska University Hospital, Gothenburg, Sweden.

### Safety surveillance for SADs

To evaluate the safety, we monitored patient samples at baseline and monthly after treatment initiation to detect adverse events, including SADs. We analyzed blood samples for complete blood counts, levels of creatinine, free thyroxin, thyroid stimulating hormone, and liver function. We analyzed urine dipstick tests to determine levels of glucose, ketones, erythrocytes/hematuria, pH, protein, nitrite, and leukocytes. When adverse events were suspected, we monitored symptoms or signs of AITD or non-thyroid SADs according to current recommendations for ALZ treatment. The time of SAD diagnosis was set to the first sign of either a deviation in laboratory measures or clinical symptoms of SAD. We classified AITD subtypes according to classes defined in a previous study of ALZ-induced AITD (23). These classes included Graves' disease, fluctuating Graves' disease, autoimmune thyroiditis, transient thyroiditis, and TRAb-positive hypothyroidism.

Non-thyroid SADs were defined as any autoimmunity other than thyroid that occurred during follow-up, including the following:

- ITP is defined as an isolated low platelet count, with normal bone marrow and no other cause of low platelets (24).
- Secondary autoimmune neutropenia is defined as a reduction in the absolute number of circulating neutrophils, below the normal threshold for age (25).
- Warm antibody hemolytic anemia is defined as auto-Abs that attach to and destroy red blood cells at normal or above normal body temperature (26).
- Autoimmune nephropathy with Goodpasture syndrome is defined as GBMAbs detected in blood or kidneys or the presence of blood and protein in the urine (27).
- Autoimmune hepatitis is defined as an abnormal liver function test result in the absence of viral hepatitis serology; liver damage and cholestasis were not induced by alcohol or toxins; ANA and SMA positivity were not required (28).

The temporal relationship between ALZ-induced auto-Abs and a subsequent SAD diagnosis was determined monthly.

### Other autoimmune diseases and smoking as risk factors for SADs

We retrospectively collected information on the presence of other autoimmune diseases besides MS, as well as previous and current smoking habits, at the baseline and after initiating ALZ treatment. For most patients, we reviewed local medical records (*n* = 77). For the remaining patients (*n* = 47), we assessed information in medical records from other healthcare regions through the “National Patient Overview,” a cooperative service for healthcare providers in Sweden.

### Clinical and MRI outcome measures

Disability was clinically assessed with the Expanded Disability Status Scale (EDSS) (29) at baseline, at 6 and 12 months, and then annually, throughout follow-up. Confirmed disability worsening (CDW) was defined as an increase of at least 1 point in the EDSS score compared to the baseline, sustained for two follow-up visits separated by an interval no less than < 6 months, and was achieved at 1.5 points when the baseline EDSS score was = 0, and then by 0.5 points when the baseline EDSS score was ≥ 5.5. We also recorded clinical relapses, defined as a new or worsening neurological disturbance episode that lasted for at least 24 h (30). At the same time points, a cerebral MRI was performed, with a 1.5 or 3.0 Tesla machine at a thickness of 3 mm according to a standard protocol for MS. We acquired T1- and T2-weighted sequences, performed fluid-attenuated inversion recovery, and acquired T1 sequences after a standard dose of intravenous gadolinium (31). The result was termed no evidence of disease activity-3 (NEDA-3) when the assessments showed no evidence of new and/or enlarged T2 lesions or gadolinium-enhanced lesions, no disability worsening, or relapses (32).

### Statistical analyses

Data were analyzed with descriptive statistical analyses. Continuous variables are expressed as the median and range. Categorical variables are expressed as frequency and percentage. To compare continuous variables, we performed the non-parametric Mann–Whitney U-test. To compare categorical variables, we performed the chi-square test. To investigate the ability of the presence of thyroid auto-Abs at baseline, at 6, 12, and 24 months to predict AITD, Cox proportional hazards regression models were performed, and hazard ratios (HRs) and 95% confidence intervals (CIs) were calculated. Binary logistic regression was performed to evaluate the impact of baseline TRAb detection on the development of AITD. The probability of developing SADs or AITD was estimated with Kaplan–Meier plots, and the results are expressed as fractions with 95% CI. The Log-Rank Mantel–Cox tests were performed to determine significant differences between the Kaplan–Meier curves. Patients with AITD at the baseline were included in the descriptive report but were excluded from predictive analyses regarding AITD development during follow-up. SPSS version 28.00 (IBM, NY, US) and GraphPad Prism 9.3.1 (GraphPad Inc., California, USA) were used for statistical analyses. All tests were two-sided, with a significance threshold of a *p* < 0.05.

## Results

We included a total of 124 (74 female subjects) patients with RRMS, of which three patients participated in CARE-MS I (4) and its extension study TOPAZ (22). A total of 77 patients were recruited from the MS Center of Sahlgrenska University Hospital, Gothenburg, and 47 patients from the “Immunomodulation and Multiple Sclerosis Epidemiology” study, at the Karolinska Institute, Stockholm, Sweden.

Most patients (*n* = 85, 69%) switched to ALZ from other highly effective disease-modifying therapies (natalizumab, rituximab, and fingolimod), 18 (14%) patients switched from a moderately effective therapy (dimethyl fumarate, teriflunomide, interferon beta, and glatiramer acetate), and 21 (17%) patients were treatment-naive. Patients were switched to ALZ when a disease breakthrough occurred during an ongoing disease-modifying therapy (*n* = 57), when JC-virus was detected in patients treated with natalizumab (*n* = 30), when adverse events occurred (*n* = 12), or for other reasons (e.g., family planning and patient request; *n* = 4). The median follow-up time was 4.5 years (ranging from 1.3 to 11.2). Baseline patient demographic and clinical characteristics are presented in Table 1.

**Table 1:** Clinical and demographic characteristics of patients with RRMS treated with ALZ.

**Characteristics**	**RRMS population (*n* = 124)**
Sex, female/male, *n* (%)	74 (60)/50 (40)
Age (years), median (range)	33.4 (21.1–58.3)
Time from first symptoms of RRMS (years), median (range)	5.2 (0.1–21.9)
Time with confirmed RRMS diagnosis (years), median (range)	3.25 (0–20.4)
EDSS at baseline, median (range)	2 (0–6)
No. of relapses in year prior to ALZ, median (range)	1 (0–4)
No. of DMTs prior to ALZ, median (range)	2 (0–5)
**Last DMT prior to ALZ**, ***n*** **(%)**	
None (i.e., treatment naive)	21 (17)
Natalizumab	61 (50)
Fingolimod	12 (10)
Rituximab	12 (10)
Dimetylfumarate	7 (5)
Teriflunomide	6 (4)
Beta interferons	4 (3)
Glatiramer acetate	1 (1)
**Reason for switching to ALZ**, ***n*** **(%)**	
Disease breakthrough during ongoing DMT	57 (46)
JC-virus positivity during natalizumab treatment	30 (24)
Side effects	12 (10)
Not applicable (i.e., treatment naive)	21 (17)
Other	4 (3)
**Autoimmune disease, other than MS, at baseline**, ***n*** **(%)**	
AITD	4 (3)
Type 1 diabetes	1 (1)
Psoriasis	1 (1)
Rheumatoid arthritis	1 (1)
**Smoking status**, ***n*** **(%)**	
Never smoked	103 (83)
Previous/current smoker	21 (17)
**No. of cycles of ALZ treatment**, ***n*** **(%)**	
1	124 (100)
2	121 (98)
3	21 (17)
4	1 (1)

EDSS, Expanded Disability Status Scale; ALZ, alemtuzumab; RRMS, relapsing-remitting multiple sclerosis; DMT, disease-modifying therapies; MS, multiple sclerosis; AITD, autoimmune thyroid disorder.

### Risk of ALZ-induced AITD and the appearance of thyroid auto-Abs

**Figure 1:**
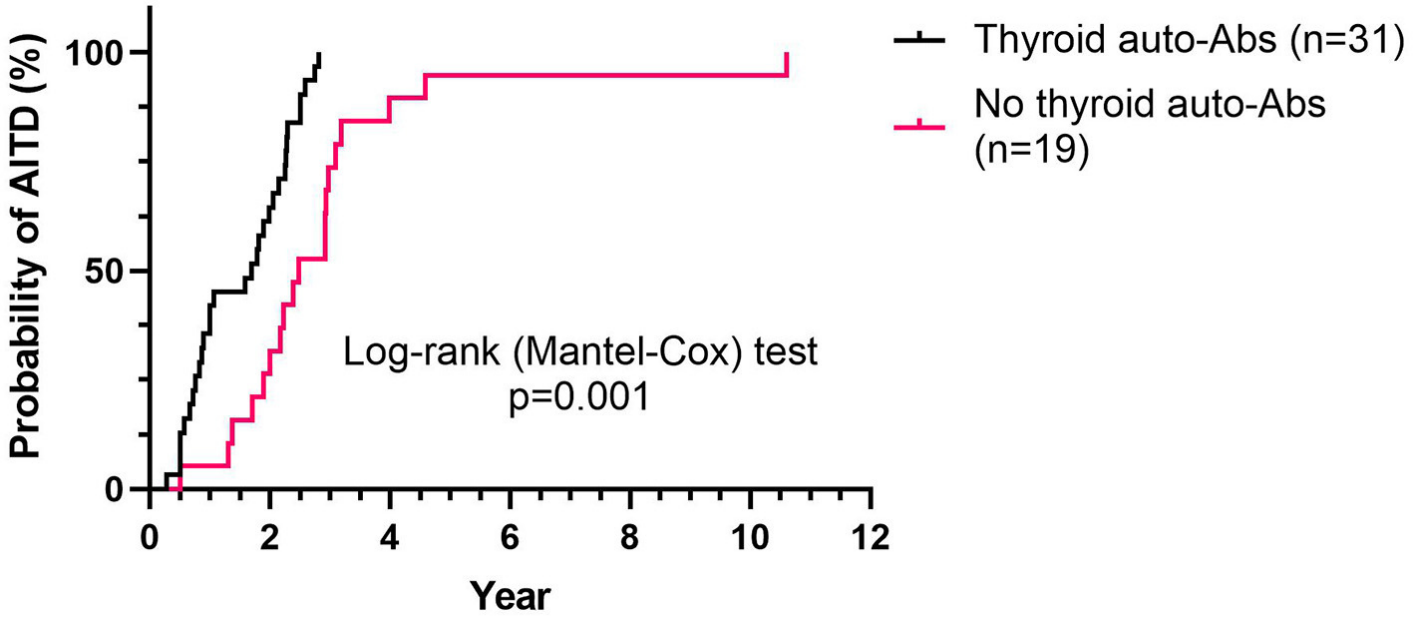
Time to AITD diagnosis, relative to thyroid auto-Abs status. Kaplan–Meier plot shows the probability of developing AITD at the indicated time (years), according to the presence (*n* = 31) or absence (*n* = 19) of thyroid auto-Abs, among patients that developed an AITD during follow-up (*n* = 50). AITD, autoimmune thyroid disorder; auto-Abs, autoimmune antibodies.

**Figure 2:**
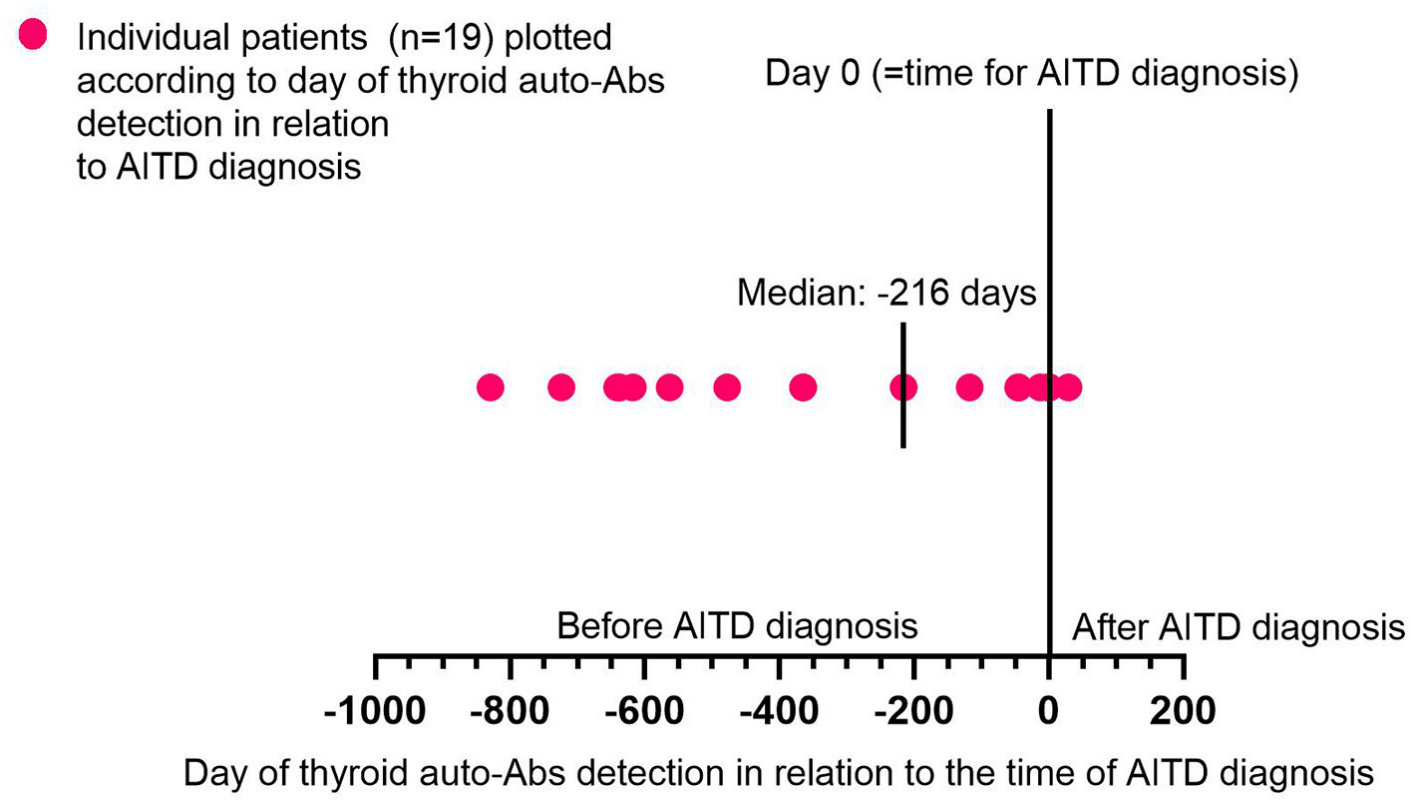
Time of thyroid auto-Abs detection in relation to AITD diagnosis. Graph shows individual patients (*n* = 19) plotted according to the day of thyroid auto-Abs detection in relation to the time of AITD diagnosis. The x-axis shows the timeline for days before (–) and after (+) the day of AITD diagnosis (=day 0). On the graph is also the median time for thyroid auto-Abs detection in relation to the AITD diagnosis highlighted. Auto-Abs, autoimmune antibodies; AITD, autoimmune thyroid disorder.

During a median follow-up of 4.5 years (ranging from 1.3 to 11.2), 50 patients (40%) developed ALZ-induced AITD (*n* = 43 Graves' disease, *n* = 5 autoimmune thyroiditis, and *n* = 2 transient thyroiditis). Thyroid auto-Abs (TgAb, TPOAb, or TRAb) were detected at some point during the study in 31 patients diagnosed with AITD. The median time from the baseline to AITD was 2.0 years (ranging from 0.3 to 10.6). AITD was diagnosed significantly earlier among patients with thyroid auto-Abs compared to those without thyroid auto-Abs (*p* < 0.0001; Figure 1). The prevalence of thyroid auto-Abs at baseline and at 6, 12, and 24 months of follow-up is presented in Table 2. In the subgroup of patients (*n* = 51) that were followed with plasma samples every 3 months, 27 patients developed ALZ-induced AITD, and 19 of them had detectable thyroid auto-Abs. All, except one, showed detectable thyroid auto-Abs before AITD diagnosis. In median thyroid auto-Abs were detectable 216 days before AITD diagnosis in this subgroup of patients (range −829–29 days) (Figure 2).

**Table 2:** Prevalence of auto-Abs and Cox proportional hazards for thyroid auto-Abs at the baseline and at 6, 12, and 24 months of follow-up.

**Auto-Ab**	**Baseline**			**6 mon**.			**12 mon**.			**24 mon**.		
	**% (** ***n*** **/total)**	**HR (95% CI)**	***p*** **-value**	**% (** ***n*** **/total)**	**HR (95% CI)**	***p*** **-value**	**% (** ***n*** **/total)**	**HR (95% CI)**	***p*** **-value**	**% (** ***n*** **/total)**	**HR (95% CI)**	***p*** **-value**
TRAb	5 (6/114)	0.98 (0.3–3.2)	0.98	3 (2/78)	0.76 (0.1–5.8)	0.79	14 (14/102)	2.9 (1.5–5.9)	**0.002**	22 (16/73)	4.6 (2.2–9.3)	**< 0.001**
TgAb	3 (3/115)	0.55 (0.07–4.0)	0.55	6 (5/85)	1.8 (0.62–5.05)	0.28	15 (15/102)	2.7 (1.3–5.5)	**0.006**	20 (15/78)	2.3 (1.2–4.6)	**0.013**
TPOAb	3 (3/115)	1.2 (0.2–9.0)	0.84	5 (4/86)	2.1 (0.6–6.9)	0.24	18 (18/102)	3.6 (1.8–7.0)	**< 0.001**	23 (18/78)	2.6 (1.3–5.2)	**0.005**
GADab	1 (1/115)			2 (2/85)			1 (1/103)			1 (1/78)		
GBMAb	3 (3/115)			0 (0/85)			0 (0/102)			0 (0/78)		
ANA	12 (13/112)			10 (8/77)			10 (10/101)			10 (7/70)		
SMA	4 (5/112)			4 (3/77)			3 (3/101)			3 (2/70)		
AMA	0 (0/112)			0 (0/77)			0 (0/101)			0 (0/70)		

Auto-Ab, autoimmune antibody; HR, hazard ratio; CI, confidence interval; mon., months; TRAb, thyrotropin receptor Ab; TgAb, thyroglobulin Ab; TPOAb, thyroperoxidase Ab; GADAb, glutamic acid decarboxylase Ab; GBMAb, anti-glomerular basement membrane Ab; ANA, antinuclear Ab; SMA, smooth muscle Ab; AMA, antimitochondrial Ab; LKM, liver kidney microsomal antigen. Bold values indicate statistical significance.

At baseline, nine patients had thyroid auto-Abs. Out of six patients with TRAb positive at the baseline, three developed AITD during follow-up. The odds ratio for AITD was 1.51 (95% CI, 0.29–7.84) for patients with TRAb at baseline compared to those without TRAb. The HRs for TRAb to predict AITD at 12 and 24 months were 2.9 (95% CI, 1.5–5.9) and 4.6 (95% CI 2.2–9.3), respectively (Table 2). At 24 months of follow-up, 27 patients (including those with and without AITD diagnosis) were positive for TRAb, TgAb, or TPOAb, and of these, 93% (25/27) had/ or later developed AITD during follow-up. In contrast, only 30% (15/51) of patients without thyroid auto-Abs had/ or later developed AITD (*p* < 0.0001) during follow-up.

### Treatment of ALZ-induced AITD

Alemtuzumab-induced AITD was mostly treated with an anti-thyroid drug combined with levothyroxine (i.e., block-and-replace therapy), but one-third of the patients required surgery (Table 3).

**Table 3:** Treatment for ALZ-induced AITD.

**Treatment**	**AITD population, *n* = 50 *n* (%)**
No treatment	2 (4)
Levothyroxine	7 (14)
High-dose anti-thyroid drug combined with levothyroxine: i.e., block-and-replace therapy	18 (36)
Thyroidectomy	17 (34)
High-dose anti-thyroid drug	2 (4)
Radioactive iodine	1 (2)
Data missing	3 (6)

ALZ, alemtuzumab; AITD, autoimmune thyroid disorder.

Patients diagnosed with AITD that had Graves' disease and blocking TRAbs (*n* = 3) were exclusively treated with levothyroxine alone. Conversely, patients with Graves' disease and stimulating TRAbs were mostly treated with either block-and-replace therapy or thyroidectomy.

### Risk of ALZ-induced non-thyroid SADs and the appearance of specific non-thyroid auto-Abs

At a median follow-up of 4.5 years (ranging from 1.3 to 11.2), eight patients (6.5%) had developed non-thyroid SADs. Of these, five had ITP (4%), two had neutropenia (2%), and one had warm antibody hemolytic anemia (1%). The median times from baseline to non-thyroid SADs diagnoses were 2.0 years (ranging from 1 to 4.2), 0.6 years (ranging from 0.05 to 1), and 5.5 years, respectively. The early case of neutropenia that occurred after 18 days (0.05 years) could have an etiology other than autoimmune disease. However, since it occurred after ALZ initiation, we cannot rule out a causal relationship. The second case is most likely related to ALZ. At diagnosis, one patient with ITP had auto-Abs against platelets, no patient had auto-Abs against neutrophils, and the patient with warm antibody hemolytic anemia had auto-Abs against erythrocytes.

A total of 18 patients without non-thyroid auto-Abs at the baseline were positive for non-thyroid auto-Abs during follow-up. Thus, in our patient population, 1% (*n* = 1/124) had GADAbs, 10% (*n* = 13/124) had ANAs, 3% (*n* = 4/124) had SMAs, none had AMAs (M2) and/ or LKMs, and none had GBMAbs. None of these patients developed associated non-thyroid SADs. The prevalence of non-thyroid auto-Abs detected at baseline and at 6, 12, and 24 months of follow-up is presented in Table 2.

### Treatment of ALZ-induced non-thyroid SADs

Among the five patients with ITP, three experienced spontaneous resolutions, one received high doses of intravenous immunoglobulin, romiplostim, and corticosteroids, and one received a platelet transfusion combined with corticosteroids. Both cases of neutropenia were resolved; one without treatment and one received a granulocyte-colony stimulating factor. No treatment was needed for the patient with warm antibody hemolytic anemia.

### Influence of SAD and AITD on clinical activity, magnetic resonance imaging activity, and disability

The median baseline EDSS did not significantly change after a median follow-up of 4.5 years (ranging from 1.3 to 11.2). No significant differences in baseline disability were observed between the SAD and non-SAD cohorts or between the AITD and non-AITD cohorts (Table 4). The clinical and MRI activities were not different between patients that developed SAD/AITD and those that did not develop an autoimmune disease (Table 4). At follow-up, the EDSS median was 2.0 (ranging from 0 to 7.5) in the non-SAD and non-AITD groups compared to 1.5 (0–7) in the SAD and AITD groups. However, these cohorts had similar numbers of patients with CDW during follow-up (Table 4).

**Table 4:** Influence of SAD and AITD on clinical activity, MRI, and disability, at the baseline and follow-up [median 4.5 y (ranging from 1.3 to 11.2)].

	**Cohort**						
**Parameters**	**All (*****n*** = **124)**	**Non-SAD (*****n*** = **69)**	**SAD (*****n*** = **55)**		**Non-AITD (*****n*** = **74)**	**AITD (*****n*** = **50)**	
**Baseline**							
EDSS, median (range), *n*	2 (0–6), *n* = 123^*^	2 (0–6), *n* = 68	1.5 (0–6), *n* = 55	*p* = 0.45	2 (0–6), *n* = 72	1.5 (0–6), *n* = 50	*p* = 0.45
**Follow-up**							
EDSS, median (range), *n*	1.5 (0–7.5), *n* = 116^*^	2.0 (0–7.5), *n* = 64	1.5 (0–7), *n* = 52	*p* = 0.032	2 (0–7.5), *n* = 68	1.5 (0–7), *n* = 48	*p* = 0.039
CDW, *n* (proportion, %)		11 (11/64, 17%)	5 (5/52, 10%)	*p* = 0.29	11 (11/68, 16%)	5 (5/48, 10%)	*p* = 0.42
Clinical relapse, *n* (%)		14 (20%)	12 (21%)	*p* = 1.0	14 (19%)	12 (24%)	*p* = 0.66
New or enlarged hyperintense T2 lesions on MRI, *n* (%)		22 (32%)	16 (29%)	*p* = 0.69	23 (32%)	15 (30%)	*p* = 0.84
Contrast enhancement on MRI, *n* (%)		14 (20%)	12 (21%)	*p* = 1.0	15 (20%)	11 (22%)	*p* = 1.0
NEDA-3, *n* (proportion, %)		33 (33/63, 52%)	29 (29/52, 56%)	*p* = 0.85	36 (36/67, 54%)	26 (26/48, 54%)	*p* = 1.0

* p = 0.93 comparing EDSS at baseline vs. follow-up for cohort “all”. SAD, secondary autoimmune diseases; AITD, autoimmune thyroid disorder, MRI, magnetic resonance imaging; EDSS, Expanded Disability Status Scale; CDW, clinical disability worsening; NEDA-3, no evidence of disease activity-3.

**Figure 3:**
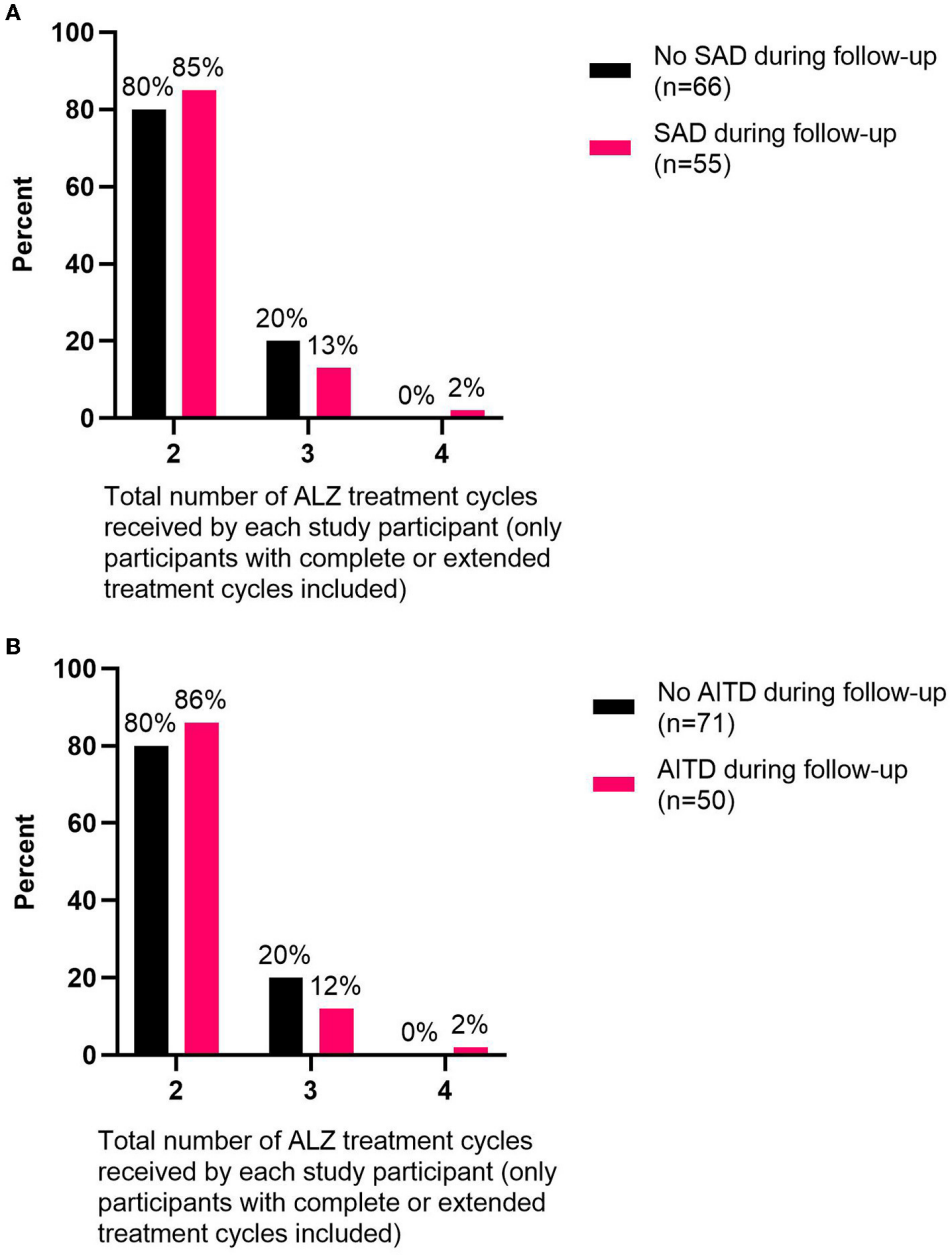
Total number of ALZ treatment cycles delivered relative to the percent of participants with/without autoimmune disorders during follow-up. The numbers of ALZ treatment cycles are shown for **(A)** study participants (only participants with complete or extended treatment cycles included) that developed SADs (*n* = 55) or did not develop SADs (*n* = 66) during follow-up and **(B)** study participants (only participants with complete or extended treatment cycles included) that developed AITDs (*n* = 50) or did not develop AITDs (*n* = 71) during follow-up. ALZ, alemtuzumab; SAD, secondary autoimmune disorder; AITD, autoimmune thyroid disorder.

The number of ALZ treatment cycles did not impact the risk of developing SAD (OR: 1.1, 95% CI, 0.49–2.46) or AITD (OR: 0.97, 95% CI, 0.42–2.26) during follow-up (Figures 3A, B).

### Smoking and other autoimmune diseases as risk factors for SADs

At baseline, 21/124 (17%) patients were previous or current smokers. Of these, 14 developed one or more SADs (67%, 14/21) during follow-up. In contrast, only 40% (41/103) of patients that had never smoked developed one or more SADs during follow-up (*p* = 0.031). During follow-up, AITD developed in approximately half of the previous/current smokers (52%, 11/21), compared to 38% (39/103) that had never smoked (*p* = 0.32).

In addition to MS, 7/124 (6%) patients reported a diagnosis of another autoimmune disease at the baseline. Of these, two (28%) developed SADs and both developed AITD after initiating ALZ treatment. In contrast, among patients with no other autoimmune disease at baseline, 45% (53/117) developed SADs (*p* = 0.46) and 41% (48/117) developed AITD (*p* = 0.7).

## Discussion

In this nationwide consecutive series of patients with RRMS that initiated ALZ, we observed that SADs and AITD developed in 24 and 21% of patients, respectively, at 24 months, and in 44 and 40% of patients, respectively, during a median follow-up of 4.5 years, consistent with previous reports (4, 5, 7, 8). Thyroid auto-Abs were detected in 62% of patients with AITD, and the presence of TRAbs at the baseline increased the risk of AITD by 50%. HR for the presence of each thyroid auto-Ab and for subsequent clinical AITD increased during follow-up and was 4.6 at 24 months for TRAb. Patients that developed thyroid auto-Abs had a significantly higher risk of AITD, and they experienced a significantly earlier AITD onset compared to patients without thyroid auto-Abs. In contrast, non-thyroid SADs were not associated with the development of non-thyroid auto-Abs.

In our study population, the different specific thyroid auto-Abs were relatively evenly distributed at baseline. This finding contrasted with findings from previous studies (17, 18, 33). Compared to our findings, two of these studies showed a higher prevalence of TPOAbs and TgAbs (18, 33), and a third study showed a slightly higher proportion of TRAb positivity, before initiating ALZ treatment (17). Data from the National Health and Nutrition Examination Survey III (34) also show a higher prevalence of TPOAbs (11.3%) and TgAbs (10.4%) in the general disease-free population than those observed at baseline in our study population (3% each), which may be due to the younger age of the MS population. In the general population, TPOAbs were associated with thyroid disease, but TgAbs alone (in the absence of TPOAbs) were not (34). In contrast, the prevalence of TRAb is close to zero in healthy persons (35) and their presence seems to be crucial in the pathogenesis of AITD (36).

The prevalence of ANA in our study population was 12% at baseline and it remained essentially unchanged during follow-up. This elevated prevalence of ANA was consistent with that found in a previous report (37), and it was higher than the prevalence found in the general population (38). Consistent with previous surveys, we did not find an elevated risk of systemic lupus erythematosus during follow-up (37). Some previous studies found that ANAs occurred at a higher frequency among patients with MS than among controls, probably reflecting an autoimmune predisposition supported by the increased risk of other autoimmune diseases reported in MS populations (38).

Our findings suggested that screening for thyroid-Abs before initiating ALZ might improve the selection of patients appropriate for ALZ treatment. At the baseline, nine of our patients had thyroid auto-Abs, but only those with TRAbs had a significantly higher risk of developing AITD. This association was consistent with the results of a previous study (17), which showed that TRAbs (measured with custom-made assays) could be detected prior to any changes in thyroid function in up to one-third of patients with post-ALZ AITD. Moreover, the presence of TRAbs prior to ALZ treatment was strongly predictive of subsequent AITD. In contrast, patients with TPOAb and TgAb positivity at the baseline did not show an increased risk of clinically manifesting AITD at follow-up. Similar results were reported in a previous study, which concluded that TPOAb alone had a limited value in risk stratification for post-ALZ AITD (33).

Thyrotropin receptor antibodies are considered pathognomonic and pathologic, and they were detected in the vast majority of patients with Graves' disease (36, 39). However, the functional consequence of TgAbs remains unclear because they do not cause thyroid cell destruction (39). In contrast, TPOAb may be cytotoxic to thyrocytes in autoimmune thyroiditis (i.e., Hashimoto's thyroiditis), but their role has not been established in Graves' disease (40). In patients treated with ALZ, Graves' disease was the most common AITD (41). This finding might explain why, among patients treated with ALZ, the role of TRAb in the risk of thyroid disease was more coherent and predictive than the roles of other thyroid auto-Abs.

The majority of patients with AITD in our study population were diagnosed with Graves' disease and they were treated according to standard procedures as previously reported in a meta-analysis (41). However, opinions diverge on the severity of ALZ-induced thyroid disease. Some authors have suggested that the disease course was more favorable in ALZ-induced Graves' disease than in conventional Graves' disease. Indeed, the majority of patients with ALZ-induced Graves' disease were successfully managed with medical treatment alone (anti-thyroid drug and/ or levothyroxine) or the disease spontaneously remitted (42). In contrast, other authors have suggested that ALZ-induced Graves' disease has unique, complex features, with an unpredictable, fluctuating course (23, 33), which frequently requires definitive treatment (i.e., radioactive iodine or thyroidectomy) (33, 41).

The incidence of ALZ-induced SADs other than AITD was considerably lower than the incidence of AITD. ITP was the second most frequent SAD; it represented 1% of SAD cases. These findings were consistent with those from the pivotal (4, 5) and extension studies (7, 8), on ALZ. Moreover, we did not find any of the autoimmune disorders described in post-marketing reports of ALZ, e.g., autoimmune hepatitis, primary biliary cholangitis, or hemophagocytic lymphohistiocytosis (9, 43). Importantly, non-thyroid SADs were not associated with the development of non-thyroid auto-Abs. Finally, we did not detect any signs of auto-Abs associated with the rare autoimmune conditions that prompted the new indication and safety monitoring for ALZ (44, 45).

It was suggested that changes following ALZ, during the immune reconstitution period (i.e., the rapid repopulation of immature B lymphocytes and homeostatic T lymphocyte proliferation), might be involved in the development of AITD (1, 14, 46, 47). It remains unclear whether these changes are connected to the beneficial effects of ALZ in RRMS. We found that patients that had developed a SAD or AITD had slightly lower EDSS scores at follow-up compared to those that did not develop a SAD. However, this finding was not supported by reductions in the rates of relapse or lesion formation on MRIs. In contrast, a previous study showed that both disability and disease activity were reduced in patients with AITD post-ALZ compared to patients without AITD (48). Estimating disability with EDSS at specific time points is less reliable compared to CDW due to intra- and inter-rater variability and to the short-term impact of relapses (12, 49, 50). Thus, the differences we found in EDSS should be interpreted with caution because they were not supported by CDW or NEDA-3.

The risk of developing a SAD was higher among current and previous smokers than among patients that had never smoked, but the risk was not affected by the presence of an autoimmune disease other than MS at baseline. Previous studies have shown that both tobacco smoking and autoimmunity were associated with several autoimmune diseases (51–54), including MS (55). However, reports on the risk of ALZ-induced autoimmunity in smokers have shown conflicting results. One previous study reported no significant association between baseline smoking status and AITD (18); conversely, another study demonstrated an odds ratio of 3.05 for AITD in patients with a smoking history at the baseline (56). Smoking was previously shown to have a broad immunologic impact. Smoking altered T- (57) and B-lymphocyte functions (58), upregulated complement factors (59), and modified gene expression (60). All or some of these effects may be important in the development of SADs post-ALZ. Thus, it would be reasonable to consider smoking a potential risk factor for SAD development post-ALZ and to incorporate this potential association in the counseling process prior to treatment.

This study had some limitations. First, although this study was a nationwide survey, only a limited number of patients were treated with ALZ in Sweden. Second, most patients received two cycles of ALZ, but three patients received only one cycle and were followed for a shorter time (the shortest follow-up time was 1.3 years). Thus, because post-ALZ SADs mostly develop within 2–3 years from the baseline, we may have missed a few isolated cases of SAD. Third, we conducted a retrospective review of medical records to obtain information on the association between AITD or non-thyroid SADs and smoking habits or concurrent autoimmune disorders other than MS in the study population. The retrospective nature of this review might have introduced the risk of unmeasured bias, and other data sources than medical records might have improved data accuracy. Furthermore, the prospective observational study design makes dropouts inevitable. Fourth, there was a 3-month interval between auto-Ab tests compared to monthly safety tests, including those for SADs. This difference would increase the likelihood of an early diagnosis of SADs compared to early detection of a prior positive auto-Ab test. However, the impact of this difference seemed limited since almost all patients in the frequently monitored subgroup developed thyroid disease after the detection of auto-Abs. Finally, only a few patients developed non-thyroid SADs. However, the results were consistent and probably reflect immune mechanisms for ALZ-induced non-thyroid SADs that are different from the mechanisms associated with thyroid autoimmune diseases.

In conclusion, this national study confirmed that patients treated with ALZ for RRMS had a high risk for SADs dominated by AITDs. We showed that thyroid auto-Abs often preceded the development of thyroid disease. This finding, concerning essentially TRAbs, can probably be used to identify individuals at high risk of AITD, before initiating and during ALZ treatment. We also confirmed that tobacco smoking increased the risk of SADs. In contrast, the risk for non-thyroid SADs was low, and monitoring non-thyroid auto-Abs did not seem to provide any additional information for predicting non-thyroid SADs.

## Data availability statement

The raw data supporting the conclusions of this article will be made available by the authors, without undue reservation.

## Ethics statement

The study involved human participants and was reviewed and approved by the Regional Ethics Review Board in Gothenburg, Sweden (Reference number 460–13). The Immunomodulation and Multiple Sclerosis Epidemiology study was approved by the Regional Ethics Review Board in Stockholm, Sweden (Reference number 2011/641-31/4). The patients/ participants provided their written informed consent to participate in this study. The study conformed with the Code of Ethics of the World Medical Association (Declaration of Helsinki) (61).

## Author contributions

SS: data analysis and interpretation, data acquisition, and manuscript drafting. LN and MA: data acquisition and critical revision of the manuscript for intellectual content. FA: data acquisition, method development, and critical revision of the manuscript for intellectual content. IK and TO: study concept and design, data acquisition, and critical revision of the manuscript for intellectual content. CM: study concept and design, data acquisition, method development, and critical revision of the manuscript for the intellectual content. JL: study concept and design, critical revision of the manuscript for the intellectual content, manuscript drafting, study supervision, and funding acquisition. All authors contributed to the article and approved the submitted version.
